# Modelling biochemical oxygen demand using improved neuro-fuzzy approach by marine predators algorithm

**DOI:** 10.1007/s11356-023-28935-6

**Published:** 2023-08-02

**Authors:** Rana Muhammad Adnan, Hong-Liang Dai, Ozgur Kisi, Salim Heddam, Sungwon Kim, Christoph Kulls, Mohammad Zounemat-Kermani

**Affiliations:** 1 School of Economics and Statistics, Guangzhou University, Guangzhou, 510006 China; 2 Department of Civil Engineering, Lübeck University of Applied Science, 23562 Lubeck, Germany; 3 Department of Civil Engineering, School of Technology, Ilia State University, 0162 Tbilisi, Georgia; 4 Faculty of Science, Agronomy Department, Hydraulics Division University, 20 Août 1955, Route El Hadaik, 21024 Skikda, BP 26 Algeria; 5 Department of Railroad Construction and Safety Engineering, Dongyang University, Yeongju, 36040 Republic of Korea; 6 Department of Water Engineering, Shahid Bahonar University of Kerman, Kerman, Iran

**Keywords:** Biochemical oxygen demand, Water quality, Prediction, Neuro-fuzzy, Marine predators algorithm

## Abstract

Biochemical oxygen demand (BOD) is one of the most important parameters used for water quality assessment. Alternative methods are essential for accurately prediction of this parameter because the traditional method in predicting the BOD is time-consuming and it is inaccurate due to inconstancies in microbial multiplicity. In this study, the applicability of four hybrid neuro-fuzzy (ANFIS) methods, ANFIS with genetic algorithm (GA), ANFIS with particle swarm optimization (PSO), ANFIS with sine cosine algorithm (SCA), and ANFIS with marine predators algorithm (MPA), was investigated in predicting BOD using distinct input combinations such as potential of hydrogen (pH), dissolved oxygen (DO), electrical conductivity (EC), water temperature (WT), suspended solids (SS), chemical oxygen demand (COD), total nitrogen (TN), and total phosphorus (T-P) acquired from two river stations, Gongreung and Gyeongan, South Korea. The applicability of multi-variate adaptive regression spline (MARS) in determination of the best input combination was examined. The ANFIS-MPA was found to be the best model with the lowest root mean square error and mean absolute error and the highest determination coefficient. It improved the root mean square error of ANFIS-PSO, ANFIS-GA, and ANFIS-SCA models by 13.8%, 12.1%, and 6.3% for Gongreung Station and by 33%, 25%, and 6.3% for Gyeongan Station in the test stage, respectively.

## Introduction

Water bodies, as the most important component of all natural resources, are essential to human survival as well as the creation of food and economic growth. However, some of these natural resources (e.g., rivers, lakes, estuaries, reservoirs, and wetlands) have recently become increasingly contaminated and polluted as a result of intensive household, agricultural, and industrial human activities. An essential and, in some instances, even crucial part of ecological control is the proper estimation of organic compound pollution of aquatic ecosystems and environmental objects. In this respect, the biochemical oxygen demand (BOD), which expresses the quantity of dissolved oxygen (DO, mg) required for the oxidation of all biodegradable organic compounds in a water sample, is known as a proper candidate for capturing the biological aspect of the biological component of water quality (Ponomareva et al. 2011).

BOD is calculated based on the difference in oxygen capacity between water samples that have been placed in special airtight flasks and the same sample after a predetermined amount of time. Hence, BOD_5_ determines the 5-day incubation of water samples saturated with oxygen and supplemented with activated sludge. Normally, BOD is measured using laboratorial tests (Tegenaw et al. 2021). Despite its precise measuring advantages, direct laboratorial tests face some limitations such as the time required for analysis and substantial expenses. To deal with these shortcomings, some researchers have utilized biosensors, which are integrated instrument that can offer analytical data that is both quantitative and semi-quantitative, for reaching a safe and rapid measurement (Wang et al. 2022). Nonetheless, the measured value of BOD using the biosensors is considered as the instantaneous value that does not necessarily correlate to the conventional BOD_5_ values.

It is worth mentioning that in some specific cases, due to laboratorial restrictions, some common water quality parameters (e.g., potential of Hydrogen (pH) and electrical conductivity (EC)) could be measured without difficulties; but the same issue may not apply to BOD. Considering this fact and the abovementioned drawbacks in measuring direct values for BOD, application of indirect methods like mathematical (Sibil et al. 2014) and artificial intelligence machine learning (AI-ML) methodologies would be worthy of consideration. Having mentioned that, AI and ML techniques have proven to be effective and efficient at simulating, optimizing, and predicting hydro-environmental applications (Zounemat-Kermani et al. 2022). In essence, AI-MLs are developed based on historical datasets, trained by simple to sophisticated optimization algorithms, and make inferences in complex systems. There are various types of AI-MLs that have been successfully employed in modeling hydro-sciences and environmental applications, like artificial neural networks (ANN), extreme learning machines (ELM) support vector regression (SVR), random forest (RF), and adaptive neuro-fuzzy inference systems (ANFIS) (Yan et al. 2010; Kim et al. 2010; Dong et al. 2023).

ANFIS is categorized as a supervised network-based ML model that combines the advantages of feedforward ANNs and fuzzy inference systems (FIS). As a result, even for a highly nonlinear system, ANFIS is expected to generate very accurate predictions. It has been widely used in predicting water quality parameters in rivers (Kisi and Zounemat-Kermani 2014; Kadkhodazadeh and Farzin 2022; Almadani and Kheimi 2023). In line with the objective of this study, specifically, Table 1 summarizes the applications of ANFIS models in modeling BOD in rivers.

**Table 1:** Some applications of the ANFIS model in relation to modeling water quality based on BOD in surface waters

Reference	Model applied and aim	Input parameters	Target value(s)	Remarks
Areerachakul (2012)	Comparison of ANFIS and ANN for estimation of BOD.	Dissolved oxygen (DO), chemical oxygen demand (COD), ammonia nitrogen (NH_3_N), nitrate nitrogen (NO_3_N), and total coliform bacteria (T-coliform).	BOD	The experimental findings show that compared to the corresponding ANFIS model, the ANN model offers a higher correlation coefficient (*R* = 0.73 for ANN vs. 0.68 for ANFIS)
Ahmed and Shah (2017)	Using ANFIS to estimate BOD of Surma River, Bangladesh.	pH, alkalinity (mg/l as CaCO_3_), hardness, total solids (TS), total dissolved solids (TDS), potassium (K^+^), PO_4_^−3^ (mg/l), NO_3_^−^ (mg/l), BOD (mg/l), and DO (mg/l).	BOD	The best ANFIS model took into account all of the input parameters with R* > 0.85 for the testing set.
Solgi et al. 2017	Using wavelet transform combined with SVR and ANFIS for modeling BOD in Karun River, Iran	Dissolved oxygen (DO), monthly temperature, and river flow.	BOD	The outcomes showed that the ANFIS model with *R*^2^ = 0.828 could not achieve as good results as the SVR model with *RMSE* = 0.0338 mg/l and *R*^2^ = 0.843.
Tiwari et al. (2018)	Applying two types of ANFISs (fuzzy c-means and subtractive clustering-based, SC) to model WQI in Satluj River, India.	pH, conductivity, chlorides, dissolved oxygen (DO), 5-day biochemical oxygen demand (BOD_5_), total dissolved solids (TDS), suspended solids (SS), ammoniacal-N, nitrates, total phosphorous (TP), and fecal coliform (FC).	Water Quality Index (WQI)	The SC-ANFIS performed better in characterizing water quality in the form of WQI.
Asghari et al. (2022)	Ensemble version of ANN, SVR, and ANFIS were used to predict effluent biological oxygen BOD and chemical oxygen demand (COD) of wastewater treatment plant.	Total suspended solids (TSS), pH at the current time (*t*), and BOD and COD at the previous time.	BOD, COD	The findings suggested that using ensemble models could boost the prediction accuracy at the verification step by up to 15%.

*R = correlation coefficient

The review of the studies illustrated in Table 1 clearly shows that, in some cases, the traditional ANFIS model cannot keep up with other types of ML models such as ANNs and SVRs. Therefore, seeking out more efficient ANFIS models seems to be a worthy effort for researchers. In fact, recent studies have exemplified the superiority of integrative (hybrid) ANFIS models embedded with meta-heuristic algorithms compared with other ML models for modeling complex environmental and hydrological problems (Zounemat-Kermani et al. 2019). Several recent reviews have been reported on the superiority of integrative ANFIS models embedded with meta-heuristic algorithms in modeling water quality (Azad et al. 2019; Aghel et al. 2019). For instance, Azad et al. (2019) employed regular ANFIS, ANFIS embedded with particle swarm optimization (ANFIS-PSO), and ANFIS embedded with ant colony optimization (ANFIS-ACO) for modeling water quality at the Zayandehrood River, Iran. Based on the general evaluation of three stations, it was demonstrated that the ANFIS-PSO acted better than the other applied ANFISs and ANN.

Having mentioned the necessity for apprising integrative (hybrid) ANFIS models in modeling complex water quality phenomena in rivers, this research aims to develop and assess the potency of four integrative ANFIS models in simulating BOD in rivers. The integrative models include two traditional meta-heuristic algorithms, namely (1) genetic algorithm (GA) (Holland 1992a), (2) particle swarm optimization (PSO) (Kennedy and Eberhart 1995); a rather new algorithm, namely (3) sine cosine algorithm (SCA) (Mirjalili 2016); and a novel algorithm called (4) marine predators algorithm (MPA) (Faramarzi et al. 2020) to develop ANFIS-PSO, ANFIS-GA, ANFIS-SCA, and ANFIS-MPA. The fundamental rationale for using GA (as an evolutionary algorithm) and PSO (as a swarm intelligence-based algorithm) is their widespread success in optimizing ML models in environmental sciences; therefore, these algorithms serve as a significant benchmark for evaluating the more current SCA and new MPA algorithms. The MPA algorithm is a simple and efficient nature-inspired algorithm that mimics the predator-prey biological interaction in oceans using Brownian motion in the search domain. This algorithm has already been known as a high-performance optimizer and won the IEEE CEC competition (Faramarzi et al. 2020). Contrary to conventional algorithms such as GA and PSO, the SCA algorithm, which is classified as a stochastic algorithm, generates more than one random solution in each step of optimization. This feature improves the potency of the algorithm in the field of optimization (Mirjalili 2016).

This matter also represents and highlights the novelty of the paper conveying the coupled application ANFIS and metaheuristic optimization methodologies. To the best of the authors’ knowledge, no study has previously used MPA with ML models to simulate water quality metrics. Accordingly, the contribution of this study lies in the evaluation of various types of integrative ANFIS models in modeling BOD based on four distinct input combinations such as pH, EC, DO, COD, SS, water temperature (WT), total nitrogen (T-N), total phosphorus (T-P), and total organic carbon (TOC). In this essence, in order to achieve a comprehensive conclusion regarding the efficiency of the integrative ANFIS models, the multivariate adaptive regression spline (MARS) model — known as one of the most qualified adaptive and robust ML models — is applied to derive and determine the optimal input combinations.

## Materials and methods

### Utilized data and study area

This study used data from two water quality stations, Gongreung and Gyeongan, South Korea, for predicting BOD parameter. Gongreung Stream (longitude 126°89′E and latitude 37°67′N), which is one of first tributaries of Han River, includes Gongreung Station located at the Gongreungcheon bridge, whereas Gyeongan Stream (longitude 127°31′E and a latitude 37°44′N), which is designated and managed as a National River of South Korea since the pollution load for Gyeongan Stream on Paldang Lake reaches 16%, involves Gyeongan Station situated at Yongdam bridge, respectively.

The number of utilized data for this study reached *N* = 583 records at Gongreung Station and *N* = 690 records at Gyeongan Station. The measurement period on both stations covers from January 1, 2008, to December 31, 2021. The data of water quality parameters were downloaded from the internet webpage (http://water.nier.go.kr) of National Institute of Environmental Research (NIER) managed by Ministry of Environment (ME), South Korea.

**Fig. 1:**
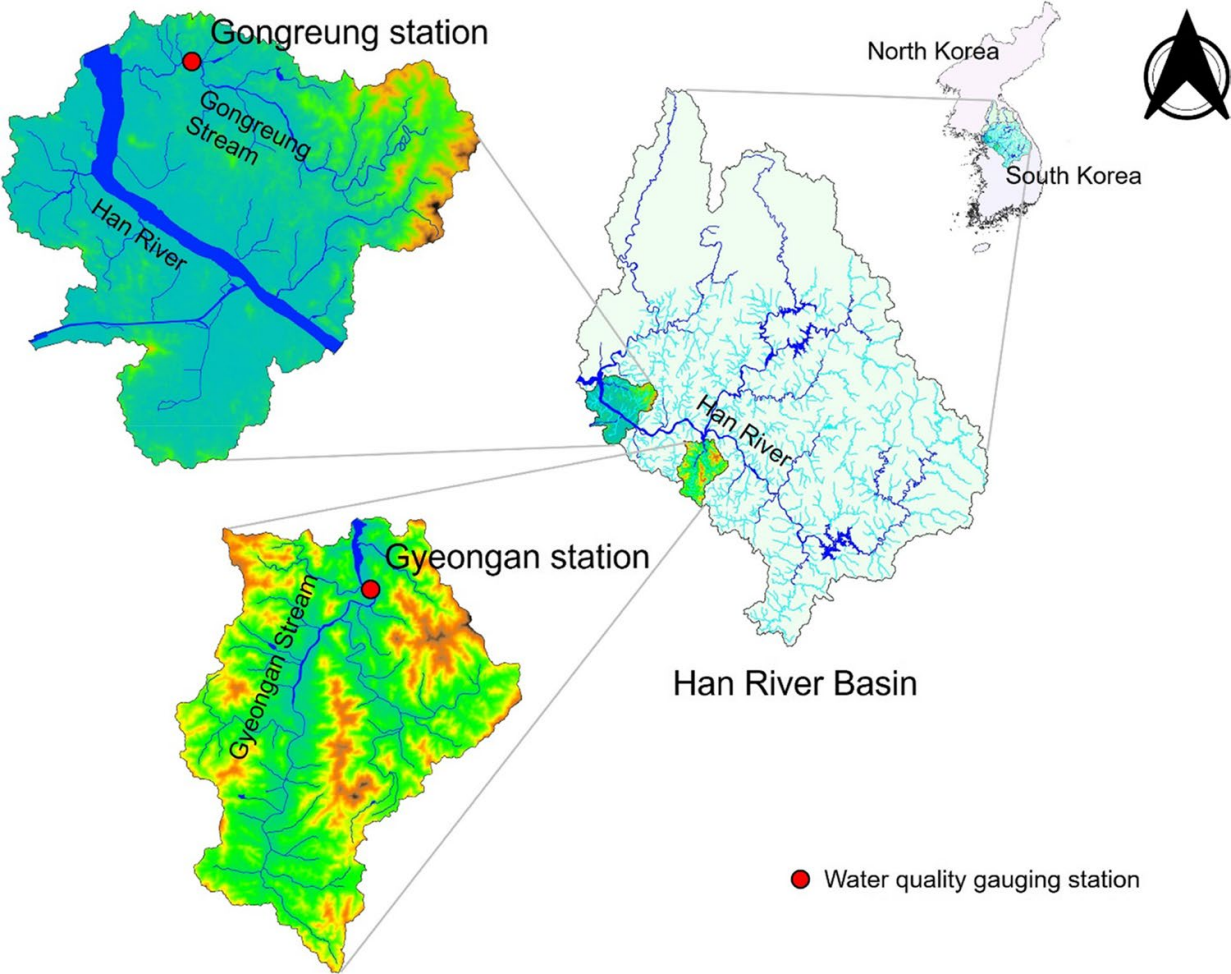
A schematic map of Gongreung and Gyeongan stations

The data were divided into two parts, training and test. The training part involved 70% of total data in both stations and the testing part included the last 30% of whole data. Figure 1 illustrates the location of the stations used in this study. Under the addressed study, the fluctuation of BOD parameter was predicted based on diverse water quality parameters including pH, EC, DO, WT, COD, SS, T-N, T-P, and TOC. Table 2 presents the brief statistical features of water quality parameters. It is visible from the Table 2 that the standard deviation values of EC and SS parameters are considerably high compared to other parameters in both stations. SS has the highest skewed distribution and distribution of BOD is far from the normal (Gaussian) distribution.

**Table 2:** Statistical properties of the water quality parameters used in the study

Station	Dataset		pH	EC	DO	WT	COD	SS	T-N	T-P	BOD
Gongreung	Training	Mean	7.94	514.91	10.98	16.86	9.53	23.88	6.28	0.22	6.26
		Min.	7.00	127.00	1.80	0.40	3.40	4.00	2.32	0.05	0.90
		Max.	9.60	2203.00	20.20	34.00	39.20	402.90	13.36	1.42	30.60
		Skewness	0.99	2.85	0.50	−0.23	2.57	8.15	0.78	3.92	1.78
		Std.dev.	0.58	247.56	2.95	8.61	3.56	29.57	2.54	0.12	3.54
	Testing	Mean	8.09	413.23	10.59	17.57	5.75	13.72	4.53	0.12	2.94
		Min.	7.10	175.00	5.30	1.30	2.00	0.80	0.46	0.03	0.50
		Max.	9.30	1594.00	17.60	32.30	19.60	381.00	14.78	0.94	15.50
		Skewness	0.58	3.28	0.03	−0.14	1.88	10.59	1.44	4.85	2.49
		Std.dev.	0.46	162.84	2.35	8.02	2.97	30.90	2.39	0.09	2.39
Gyeongan	Training	Mean	8.12	376.30	11.12	14.66	5.90	11.40	4.95	0.09	2.55
		Min.	6.90	110.00	5.30	0.00	2.80	0.70	1.68	0.01	0.60
		Max.	9.50	642.00	17.50	29.70	13.50	168.70	12.27	0.43	10.60
		Skewness	0.86	−0.31	0.10	−0.15	1.19	5.67	0.81	1.99	1.37
		Std.dev.	0.44	103.74	2.42	8.66	1.98	15.50	2.06	0.07	1.74
	Testing	Mean	8.05	383.27	10.99	15.59	5.13	11.66	4.19	0.07	1.82
		Min.	7.50	132.00	7.20	0.60	3.20	1.00	1.77	0.02	0.50
		Max.	9.10	549.00	16.20	30.70	13.00	207.00	7.81	0.58	6.70
		Skewness	1.06	−0.61	0.36	−0.12	1.68	6.05	0.34	4.86	1.67
		Std.dev.	0.29	74.86	2.30	8.46	1.68	22.73	1.34	0.06	1.20

### Multivariate adaptive regression splines

The MARS machine learning model was proposed by Friedman (1991). MARS can be considered as a tree based (TB) machine learning algorithm, and it uses the idea of dividing the dataset space into several subspace and building a spline functions (i.e., basis functions) for each subspace. The output of the MARS model is calculated as follows (Chen et al. 2022):

1 $$\hat{Y}={\beta}_0+\sum_{m=1}^M{\beta}_m{\varnothing}_m\left[x\right]\kern1em$$

**Fig. 2:**
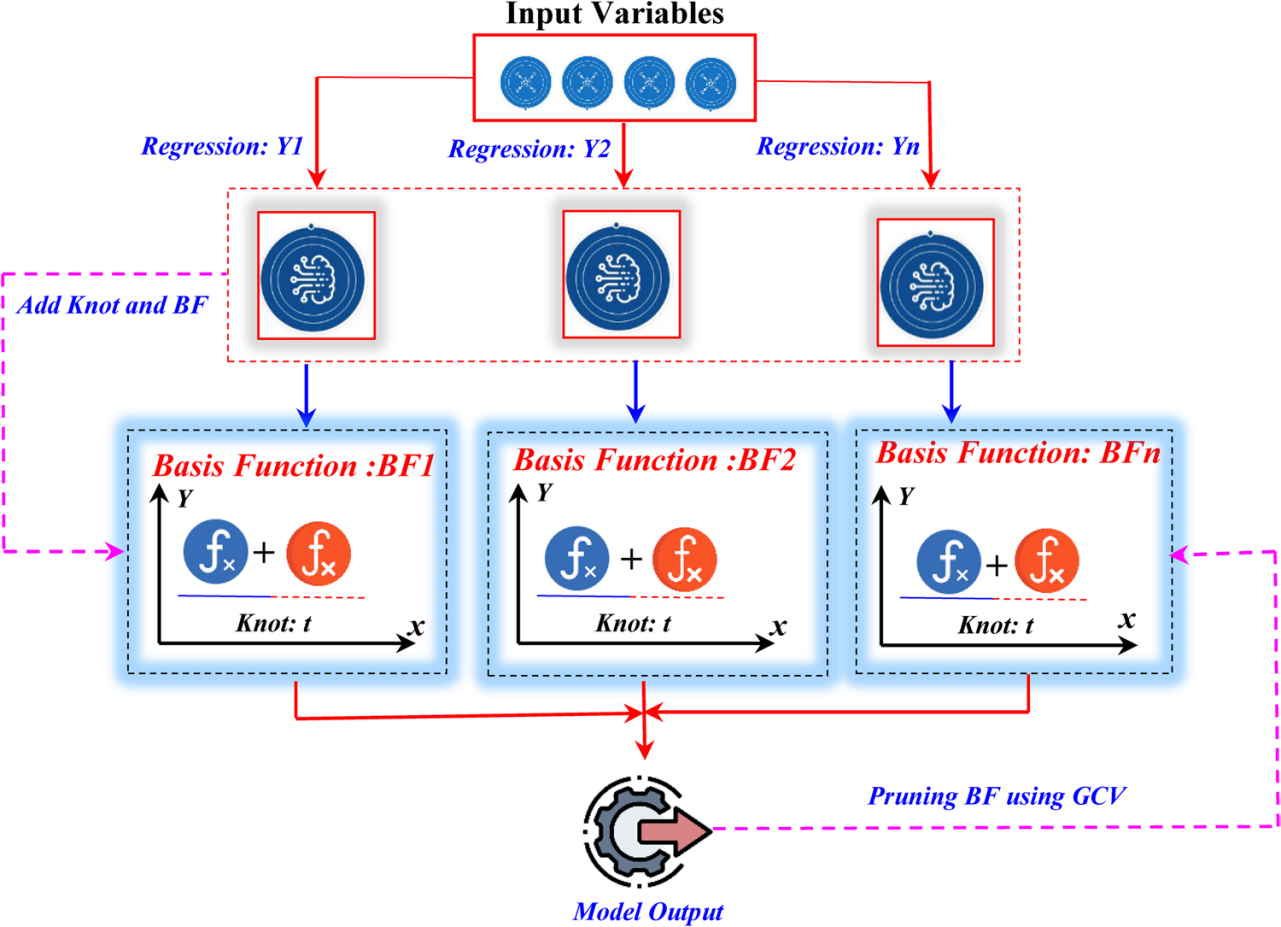
Structure of MARS

In the above equation, $\hat{Y}$ is the calculated value of the target variable, *β*_*m*_ is the constant term, *β*_*m*_ is the coefficient corresponding to the *m*th spline function, and ∅_*m*_ is the *m*th spline function. In MARS model, the breakpoint used for moving from one function to another is called the *Knots*, and it is important to note that one of the major advantages of the MARS model is its capability for searching the input variables (i.e., the independent variables) one by one which can help in avoiding any degree of interaction between the independent variables. MARS model can be developed in two different steps. First, an ensemble of basis functions (BFs) is constructed (i.e., the forward pass). During the second stage (i.e., the pruning pass), the generalized cross-validation (GCV) is adopted as a criteria for removing or deleting the BFs that have a poor contribution, and the variable importance is calculated by measuring the degree of reduction in the calculated GCV when removing each one from the independent variables of the model (Wang et al. 2023; Jin et al. 2023). Figure 2 illustrates the structure of MARS.

### Adaptive neuro-fuzzy inference system

**Fig. 3:**
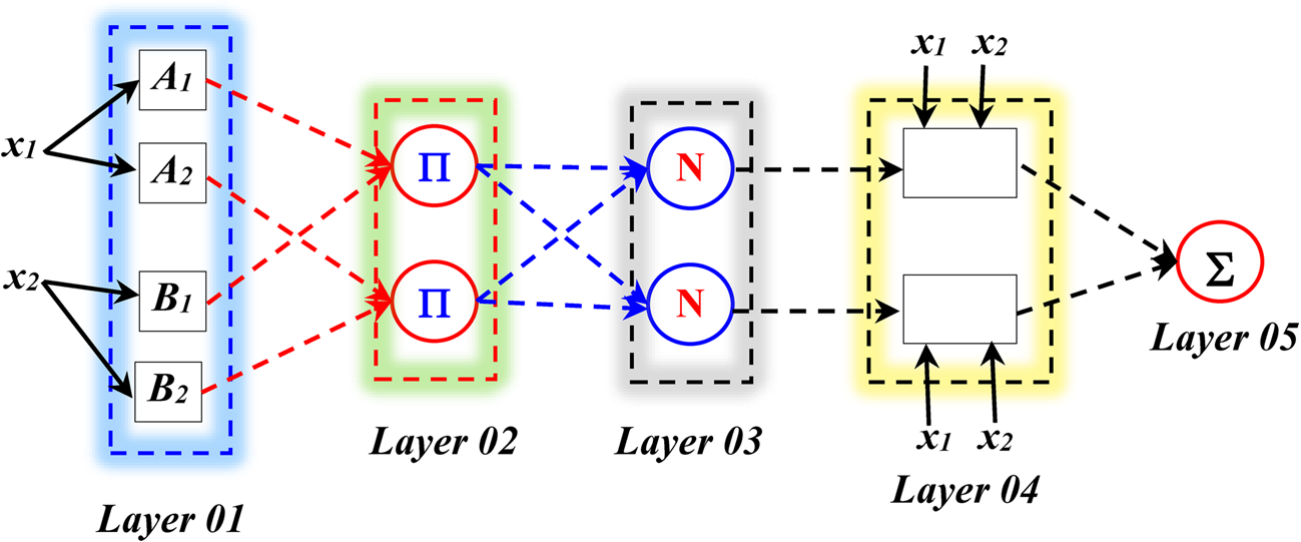
Structure of ANFIS

The ANFIS was first introduced by Jang (1993). The ANFIS model can be viewed as a multilayer feed-forward artificial neural network for which two techniques were combined for building a single model: the ANN and the fuzzy inference reasoning. ANFIS model is used for relating an ensemble of input variables *x*_*i*_ to one output variable *y* based on a nonlinear mathematical formulation. The input variables are expressed using linguistic descriptions (i.e., low, middle, high, very high, respectively), and for each linguistic terms, a membership function (MF) is adopted, i.e., the *μ*_*i*_(*x*_*i*_). The ANFIS model uses an ensemble of input/output dataset for building a fuzzy inference system (FIS), and similar to all machine learning models, there are an ensemble of updated parameters, i.e., the nonlinear MF parameters and the linear parameters of the fuzzy rules. ANFIS model has five layers, which can be briefly described as follows (Fig. 3) (Kumar et al. 2023; Sarkar et al. 2023).

#### Layer 01 (fuzzification layer)

Each node here is a square node with the following function:

2 $${O}_{1,i}={\mu}_{A_i}\left({x}_1\right),\kern5.75em i=1,2$$

3 $${O}_{1,i}={\mu}_{B_i}\left({x}_2\right),\kern5.75em i=3,4$$

where *x*_1_ and *x*_2_ are the input variables and *A*_1_ and *B*_2_ correspond to the linguistic label. The *O*_*1,i*_ can be viewed as the MF of *A*_*i*_ and *B*_*i*_. In this first layer, the parameters of the MF correspond to the premises parameters or the nonlinear parameters of the ANFIS model.

#### Layer 02 (product layer)

Each node here is a circle node labeled Π*.* Its output can be calculated as follows:

4 $${O}_{2,i}={w}_i={\mu}_{A_i}\left({x}_1\right)\bullet {\mu}_{B_i}\left({x}_2\right),\kern5.75em i=1,2,3,4$$

#### Layer 03 (normalization layer)

Each node here is a circle node labeled N. Its output can be calculated as follows:

5 $${O}_{3,i}={\overline{w}}_i=\frac{w_i}{w_1+{w}_2+\dots {w}_i}\kern5em i=1,2,3,4$$

#### Layer 04 (defuzzification layer)

Each node here is a square node with following function:

6 $${O}_{4,i}={\overline{w}}_if={w}_i\left({p}_i{x}_1+{q}_i{x}_2+{r}_i\right)\kern4.5em i=1,2,3,4$$

where *w*_*i*_ corresponds to the output of layer 3 and {*p*_*i*_*, q*_*i*_*, r*_*i*_} are the parameter of the fuzzy rules. These parameters are the consequent parameters (i.e., the linear parameters).

#### Layer 05 (output layer)

Only one node is available in this layer, and it computes the overall response of the model as the summation of all incoming signals from the previous layers as follows:

7 $${O}_{5,i}=\mathrm{final}\ \mathrm{response}={\overline{w}}_if=\frac{\sum_i{w}_i{f}_i}{\sum_i{w}_i}$$

### Genetic algorithm

**Fig. 4:**
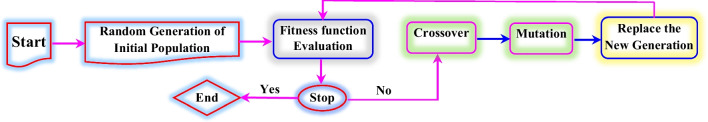
Flowchart of GA

GA is a global optimization method (Holland 1992b) broadly reported in the literature as an efficient tool for improving the performances of machine learning models. The GA is mainly inspired from the reproduction behavior and it can be achieved in four steps: reproduction, selection, crossover, and mutation (Fig. 4). The algorithm starts by randomly generating a population of individuals (i.e., chromosomes). The population is evaluated using a fitness function. Thus, the GA updates the initial population using selection, crossover, and mutation until the best solution is obtained which determines the stopping criteria (Jamali et al. 2019; Salim et al. 2019; Satrio et al. 2019).

### Particle swarm optimization

PSO is a metaheuristic algorithm based on swarm intelligence mainly inspired from the behavior of the swarm movement, i.e., bees, fish schools, and insects while searching the prey; it was developed by Kennedy and Eberhart (1995). The overall PSO algorithm can be described as follows. The individuals are called particles, and they play the role of agents, and there is a communication between the agents. 

**Fig. 5:**
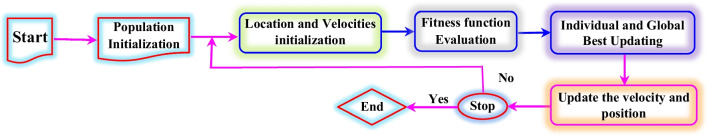
Flowchart of PSO

They form an extremely dense swarm, which cannot be dissociated. The PSO is composed from three parts (Alam et al. 2014): (*i*) particles, (*ii*) social and cognitive components of the particles, and (*iii*) velocity of the particles. The PSO is an iterative algorithm, and at each iteration, each individual (i.e., particle) is localized in a specific point (i.e., position) with a particular velocity vector; thus, each particle has both a velocity and a position. More precisely, during the training process, the velocity is updated continuously taking into account the same velocity in the previous iteration, the direction of the best position of the particle, and the best position of any other particle (Regis 2014). Each position can be considered as a probable solution; therefore, the particle is evaluated based on fitness function until the convergence condition was obtained (Fig. 5). Finally, the particle having the best fitness is then selected as the global and best solution (Ghorbani et al. 2014).

### Sine cosine algorithm

**Fig. 6:**
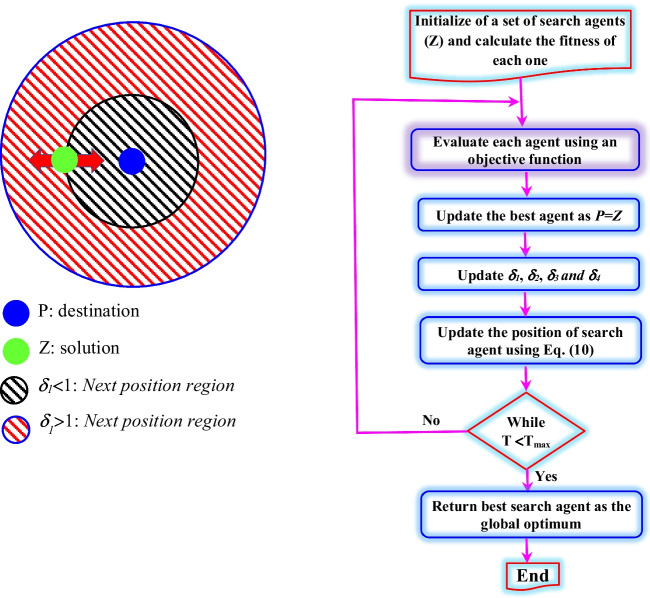
Flowchart of SCA (Mirjalili 2016)

SCA developed by Mirjalili (2016) belongs to the category of population-based optimization algorithms. The SCA algorithm starts by presenting an ensemble of possible solution, and using an objective function, the set of solution is repeatedly evaluated, and the chance for finding the best solution increases by the increase of the number of iterations. Similar to the majority of optimization algorithms, the SCA has two phases: the exploration and exploitation phases (Mirjalili 2016). The two equations presented hereafter are used for both exploration and exploitation phases (Fig. 6):

8 $${Z}_i^{t+1}={Z}_i^t+{\delta}_1\times \sin \left({\delta}_2\right)\times \left|{\delta}_3{L}_i^t-{Z}_i^t\right|,\kern1.25em$$

9 $${Z}_i^{t+1}={Z}_i^t+{\delta}_1\times \cos \left({\delta}_2\right)\times \left|{\delta}_3{L}_i^t-{Z}_i^t\right|,\kern1.25em$$

where ${Z}_i^t$ corresponds to the actual position of the existing solution in *i*th dimension at *t*th, iteration; *δ*_1_, *δ*_2_, and *δ*_3_ are random numbers; *L*_*i*_ corresponds to the destination point’s position in *i*th dimension; and || indicates the absolute value (Mirjalili 2016). By combining the two previous equations, we can obtain the following equation:

10 $${Z}_i^{t+1}=\left\{\begin{array}{c}{Z}_i^t+{\delta}_1\times \sin \left({\delta}_2\right)\times \left|{\delta}_3{L}_i^t-{Z}_i^t\right|,\kern2.25em {\delta}_4<0.5\ \\ {}{Z}_i^t+{\delta}_1\times \sin \left({\delta}_2\right)\times \left|{\delta}_3{L}_i^t-{Z}_i^t\right|,\kern0.5em {\delta}_4\ge 0.5\kern0.5em \end{array}\right.\kern1.25em$$

where *δ*_4_ is a random number in [0, 1].

From the above equation, it is clear that the SCA needs four parameters, namely, *δ*_1_, *δ*_2_, *δ*_3_, and *δ*_4_. The *δ*_1_ is responsible for determining the exact movement direction. The *δ*_2_ is responsible for determining yet whether the movement ought to be *towards* or *outwards* the destination. The *δ*_3_ can be whether an *emphasize* (*δ*_3_ > 1) or *deemphasize* (*δ*_3_ < 1). Finally, the *δ*_4_ equally switches between the components of sine and cosine (Mirjalili 2016). The *δ*_1_ can be calculated as follows:

11 $${\delta}_1=a-t\frac{a}{T}$$

### Marine predator’s algorithm

**Fig. 7:**
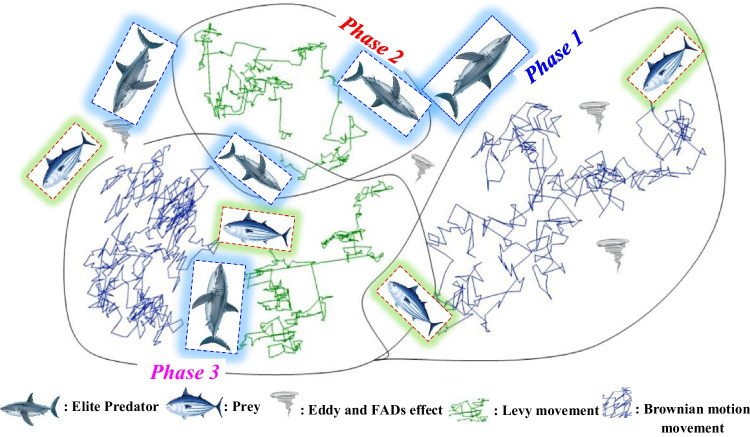
Flowchart of MPA algorithm (Faramarzi et al. 2020)

**Fig. 8:**
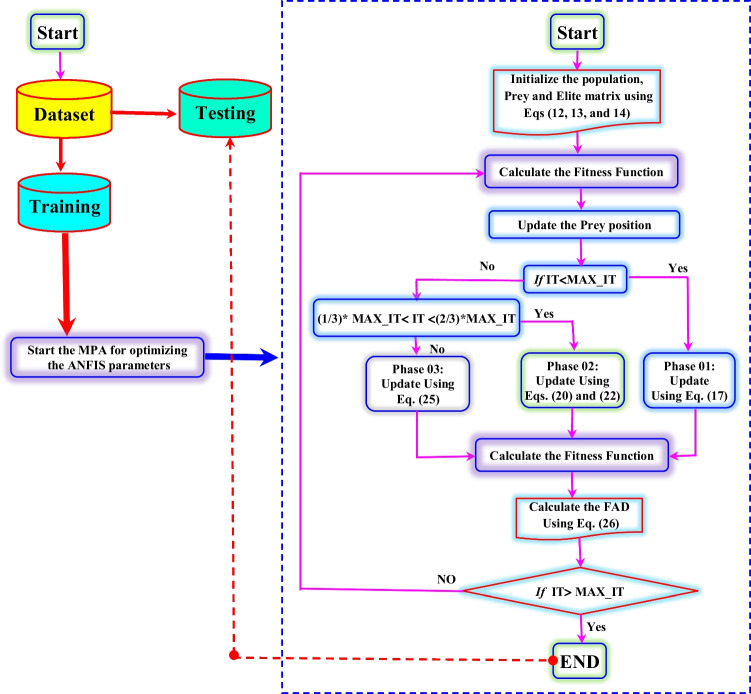
ANFIS-MPA flowchart

The MPA was introduced by Faramarzi et al. (2020), and it is based on the idea of simulating the behavior of ocean predators foraging strategy using the *Lévy* and *Brownian* movements (Fig. 7). Similar to several other population, the MPA initial candidate’s solutions should be proposed for the first iteration as follows (Fig. 8):

12 $${X}_0={X}_{\mathrm{min}}+\mathit{\operatorname{rand}}\ \left({X}_{\mathrm{max}}-{X}_{\mathrm{min}}\right)$$

where *X*_min_ and *X*_max_ correspond to the lower and upper bounds and *rand* is a uniform random vector having the scale from 0 to 1. Using the so-called *survival of the fittest theory*, an initial matrix called the *Elite* (*EL*) is constructed as follows:

13 $$EL={\left[\begin{array}{c}\begin{array}{c}\begin{array}{c}{X}_{1,1}^I\kern0.5em \\ {}{X}_{2,1}^I\kern0.5em \end{array}\\ {}\vdots \end{array}\\ {}\vdots \\ {}\vdots \\ {}{X}_{n,1}^I\ \end{array}\begin{array}{c}{X}_{1,2}^I\\ {}{X}_{2,2}^I\\ {}\vdots \\ {}\vdots \\ {}\vdots \\ {}{X}_{n,2}^I\end{array}\begin{array}{c}\cdots \kern0.5em \\ {}\cdots \kern0.5em \\ {}\vdots \\ {}\vdots \\ {}\vdots \\ {}\cdots \kern0.5em \end{array}\begin{array}{c}{X}_{1,d}^I\\ {}{X}_{2,d}^I\\ {}\vdots \\ {}\vdots \\ {}\vdots \\ {}{X}_{n,d}^I\end{array}\right]}_{n\times d}$$

The $\overrightarrow{X^I}$ is considered the top predator vector, *n* is the number of search agent, and *d* is the number of dimensions. It is important to note that the search agent terms should be attributed for both predator and prey. A second matrix called *Prey* (*PR*) having the same dimension as the *Elite* was used as a reference for updating the position of the *Elite*, and it is expressed as follows:

14 $$PR={\left[\begin{array}{c}\begin{array}{c}\begin{array}{c}{X}_{1,1}^I\kern0.5em \\ {}{X}_{2,1}^I\kern0.5em \end{array}\\ {}\vdots \end{array}\\ {}\vdots \\ {}\vdots \\ {}{X}_{n,1}^I\ \end{array}\begin{array}{c}{X}_{1,2}^I\\ {}{X}_{2,2}^I\\ {}\vdots \\ {}\vdots \\ {}\vdots \\ {}{X}_{n,2}^I\end{array}\begin{array}{c}\cdots \kern0.5em \\ {}\cdots \kern0.5em \\ {}\vdots \\ {}\vdots \\ {}\vdots \\ {}\cdots \kern0.5em \end{array}\begin{array}{c}{X}_{1,d}^I\\ {}{X}_{2,d}^I\\ {}\vdots \\ {}\vdots \\ {}\vdots \\ {}{X}_{n,d}^I\end{array}\right]}_{n\times d}$$

More importantly, it is worth to note that the MPA optimization procedure is completely governed by these two matrices. The MPA algorithm can be achieved in three phases depending on the velocity ratios and simultaneously the nature life of both the prey and predator: (*i*) the high velocity ratio (the prey faster than the predator), (*ii*) the unit velocity ratio, and (*iii*) low velocity ratio (the predator faster than the prey) (Faramarzi et al. 2020). All three steps are achieved using an important number of iterations.

The high velocity ratio (phase 1: *v* ≥ 10). This phase 1 is available during the starting of the iteration process (i.e., during the exploration), and it is characterized by the fact that the prey moves faster than the predator. The mathematical formulation is as follows:

15 $$\mathrm{While}\ IT<\frac{1}{3}\mathit{\operatorname{MAX}}\_ IT$$

16 $${\overrightarrow{STZ}}_i={\overrightarrow{R}}_B\oplus \left({\overrightarrow{EL}}_i-{\overrightarrow{R}}_B\oplus {\overrightarrow{PR}}_i\right);i=1,2,3,\dots, n.$$

17 $${\overrightarrow{PR}}_i={\overrightarrow{PR}}_i+0.5\overrightarrow{R}\oplus {\overrightarrow{STZ}}_i$$

where *IT* is the actual iteration, *MAX_IT* corresponds to the maximal number of iterations, *R*_B_ is a random number for the expression of the *Brownian* motion, *STZ* is the *stepsize*, *PR* is the prey, *R* is an uniform number between [0,1], and ⊗ is the entry wise multiplication (Faramarzi et al. 2020).

The unit velocity ratio (phase 2: *v* ≈ 1). The prey and the predator move at the same pace. This is a transition phase for which the exploration prepares to pass into the exploitation phase: the two were matters. We can see during this phase that the population is split in two equal parts: one for the exploitation (i.e., the prey) and the second half for the exploration (i.e., the predator). More precisely, if prey moves in *Lévy*, the best strategy for predator is *Brownian*. This phase can be formulated as follows:

18 $$\mathrm{While}\kern0.5em \frac{1}{3} MA{X}_{IT}< IT<\frac{2}{3} MA{X}_{IT}$$

For the 1st half of the population

19 $${\overrightarrow{STZ}}_i={\overrightarrow{R}}_L\oplus \left({\overrightarrow{EL}}_i-{\overrightarrow{R}}_L\oplus {\overrightarrow{PR}}_i\right);i=1,\dots, {~}^{n}\!\left/ \!{~}_{2}\right..$$

20 $${\overrightarrow{PR}}_i={\overrightarrow{PR}}_i+0.5\overrightarrow{R}\oplus {\overrightarrow{STZ}}_i$$

For the second half of the populations,

21 $${\overrightarrow{STZ}}_i={\overrightarrow{R}}_B\oplus \left({\overrightarrow{R}}_B\oplus {\overrightarrow{EL}}_i-{\overrightarrow{PR}}_i\right);i=\frac{n}{2},\dots, n.$$

22 $${\overrightarrow{PR}}_i={\overrightarrow{EL}}_i+0.5\bullet {\left(1-\frac{IT}{\mathit{\operatorname{MAX}}\_ IT}\right)}^{\left(2\frac{IT}{\mathit{\operatorname{MAX}}\_ IT}\right)}\oplus {\overrightarrow{STZ}}_i$$

*R*_*L*_ is a vector involving random numbers which represent Lévy movement. The multiplication of${\overrightarrow{R}}_L$ and *Prey* maps the prey movement in *Lévy manner*, and the multiplication of ${\overrightarrow{R}}_B$and *Elite* simulates the movement of predator in *Brownian manner* (Faramarzi et al. 2020).

The low velocity ratio (phase 3: *v* ≈ 0.1). This is the last phase of the optimization process having as a particularity the elevated exploitation capability (i.e., the predator is moving faster than the prey). This phase can be formulated as follows:

23 $$\mathrm{While}\kern0.5em IT>\frac{2}{3} MA{X}_{IT}$$

24 $${\overrightarrow{STZ}}_i={\overrightarrow{R}}_L\oplus \left({\overrightarrow{R}}_L\oplus {\overrightarrow{EL}}_i-{\overrightarrow{PR}}_i\right);i=1,2,\dots, n.$$

25 $${\overrightarrow{PR}}_i={\overrightarrow{EL}}_i+0.5\bullet {\left(1-\frac{IT}{\mathit{\operatorname{MAX}}\_ IT}\right)}^{\left(2\frac{IT}{\mathit{\operatorname{MAX}}\_ IT}\right)}\oplus {\overrightarrow{STZ}}_i$$

It was reported that the fish aggregating device (FAD) effects should be taken into account as it corresponds to the *local optima*, and it is expressed as follows (Faramarzi et al. 2020):

26 $${\overrightarrow{PR}}_i=\left\{\begin{array}{c}{\overrightarrow{PR}}_i+{\left(1-\frac{IT}{\mathit{\operatorname{MAX}}\_ IT}\right)}^{\left(2\frac{IT}{\mathit{\operatorname{MAX}}\_ IT}\right)}\bullet \left[{\overrightarrow{X}}_{min}+\overrightarrow{R}\right]\oplus \left({\overrightarrow{X}}_{max}-{\overrightarrow{X}}_{min}\right)\oplus \overrightarrow{U}\kern1.75em \mathrm{if}\ r\le FADs\\ {}{\overrightarrow{PR}}_i+\left[ FADs\left(1-r\right)+r\right]\left({\overrightarrow{PR}}_{r1}-{\overrightarrow{PR}}_{r2}\right)\kern13.75em \mathrm{if}\ r> FADs\end{array}\right.$$

### Proposed ANFIS-MPA

In this section, the description of the developed hybrid ANFIS-MPA is briefly presented. Similar to all optimization algorithms, the MPA is used for optimizing the ANFIS model using a fitness function (Fig. 8). ANFIS has two kind of parameters, linear and nonlinear. The nonlinear parameters, i.e., the premise parameters are available in the first layer and they correspond to the membership function parameters. The second kind of parameters are the linear parameters of the fuzzy rules available in the fourth layer. The ANFIS-MPA starts by generating a set of random population (i.e., solution) for an ensemble of *N* agents. More precisely, one ANFIS model is constructed and evaluated tacking into account its value presented for the training subset. In the next step, the fitness functions, i.e., the mean squared error (*MSE*) and the root mean square error (*RMSE*) are used for evaluating the performances of the ANFIS-MPA model. The best-obtained solution having the best fitness values is finally retained, and the testing subset is presented for the ANFIS-MPA model for the final evaluation.

## Results

In this study, the potential of four different hybrid ANFIS models was investigated in predicting BOD using different water quality parameters involving pH, EC, DO, WT, COD, SS, T-N, and T-P. Models were assessed using monthly data obtained from two stations, Gonfreung and Gyeongan, South Korea, and three commonly used statistics, *RMSE*, mean absolute error (*MAE*), and determination coefficient (*R*^2^). The formulation of these statistics is given below:

27 $$RMSE=\sqrt{\frac{1}{N}\sum_{i=1}^N{\left({\left({Y}_o\right)}_i-{\left({Y}_c\right)}_i\right)}^2}\kern2.5em$$

28 $$MAE=\frac{1}{N}{\sum}_{i=1}^N\mid {\left({Y}_o\right)}_i-{\left({Y}_c\right)}_i\mid \kern1.5em$$

29 $$\kern1em {R}^2={\left[\frac{\sum_{t=1}^N\left({Y}_o-\overline{Y_o}\right)\left({Y}_c-\overline{Y_c}\right)}{\sqrt{\sum_{t=1}^N{\left({Y}_o-\overline{Y_o}\right)}^2{\left({Y}_c-\overline{Y_c}\right)}^2}}\right]}^2\kern1.5em$$

where ${Y}_o,{Y}_c,\kern0.5em {\overline{Y}}_{c,}\ \mathrm{and}\ N$ refer to the observed, computed, mean of the observed BOD, and data length, respectively.

Table 3 reports the input combinations considered for BOD prediction. In the table, the training and testing results of MARS method for the Gongreung Station can be observed. Here, the purpose of using MARS method is to determine the best input combination. In other words, we wanted to investigate if this method can be applicable for deciding the best scenario in modeling BOD. This was checked by applying hybrid ANFIS methods to all input scenarios. First, we started with pH, EC, DO, and WT data as input because these are basic parameters in all rivers. Then, other parameters were added into the first combination so as to observe the most effective inputs to the BOD (output) parameter. From Table 3, it is seen that the accuracy of MARS generally improves by involving additional parameter except T-P input. COD seems highly effective on BOD since by involving it in the input scenarios (see 2nd input combination), the *RMSE* and *MAE* decrease from 3.350 mg/l and 2.488 mg/l to 2.072 mg/l and 1.357 mg/l and *R*^2^ increases from 0.204 to 0.715 in the test stage. However, adding T-P slightly decreases the accuracy of MARS model in BOD prediction. Among the input scenarios, the model with pH, EC, DO, WT, COD, SS, and T-N inputs offers the best performance with the lowest *RMSE* (1.950 mg/l) and *MAE* (1.322 mg/l) and the highest *R*^2^ (0.775). Among the input scenarios considered, the best statistics were underlined in the tables.

**Table 3:** Training and test statistics of the models for BOD prediction — MARS for Gongreung Station

Model inputs	Training period			Test period		
	*RMSE*	*MAE*	*R*^2^	*RMSE*	*MAE*	*R*^2^
pH, EC, DO, WT	2.727	1.919	0.407	3.350	2.488	0.204
pH, EC, DO, WT, COD	1.902	1.316	0.742	2.072	1.357	0.715
pH, EC, DO, WT, COD, SS,	1.897	1.318	0.785	2.046	1.337	0.752
pH, EC, DO, WT, COD, SS, T-N	1.869	1.307	0.803	1.950	1.322	0.775
pH, EC, DO, WT, COD, SS, T-N, T-P	1.883	1.316	0.796	2.018	1.330	0.763
*Mean*	2.056	1.435	0.707	2.287	1.567	0.642

The training and test outcomes of the hybrid ANFIS methods, ANFIS-PSO, ANFIS-GA, ANFIS-SCA, and ANFIS-MPA, are respectively provided in Tables 4, 5, 6 and 7 in predicting BOD of Gongreung Station. In all hybrid methods, the variation of error statistics is consistent and the input scenario comprising pH, EC, DO, WT, COD, SS, and T-N input parameters produces the best accuracy in training and test stages; the lowest *RMSE* and *MAE* values are 1.628 mg/l and 1.148 mg/l for the ANFIS-PSO, 1.596 mg/l and 1.113 mg/l for the ANFIS-GA, 1.497 mg/l and 1.020 mg/l for the ANFIS-SCA, and 1.403 mg/l and 0.844 mg/l for the ANFIS-MPA in the test stage. Involving COD in the inputs of the hybrid models considerably improves their accuracy in BOD prediction, for example, *RMSE* decreases from 2.901 to 1.710 mg/l for the ANFIS-PSO, from 2.835 to 1.703 mg/l for the ANFIS-GA, from 2.616 to 1.587 mg/l for the ANFIS-SCA, and from 2.457 to 1.494 mg/l for the ANFIS-MPA in the test stage. Considering all input scenarios, the *RMSE*, *MAE*, and *R*^2^ range from 2.901 mg/l, 2.410 mg/l, and 0.268 to 1.628 mg/l, 1.148 mg/l, and 0.799 for the ANFIS-PSO; from 2.835 mg/l, 2.266 mg/l, and 0.333 to 1.596 mg/l, 1.113 mg/l, and 0.803 for the ANFIS-GA; from 2.616 mg/l, 2.130 mg/l, and 0.367 to 1.497 mg/l, 1.020 mg/l, and 0.828 for the ANFIS-SCA; and from 2.457 mg/l, 2.064 mg/l, and 0.411 to 1.403 mg/l, 0.844 mg/l, and 0.843 for the ANFIS-MPA, respectively. Comparison of the best input scenarios indicates that the ANFIS-MPA model outperforms the other models in predicting BOD of Gongreung Station by respectively improving the *RMSE* accuracies by 13.8%, 12.1%, and 6.3% in the test stage compared to the ANFIS-PSO, ANFIS-GA, and ANFIS-SCA models. Average statistics also justify the superiority of the ANFIS-MPA which has the lowest *RMSE* (1.649 mg/l) and *MAE* (1.123 mg/l) and the highest *R*^2^ (0.746) followed by the ANFIS-SCA (*RMSE* 1.754 mg/l, *MAE* 1.297 mg/l, *R*^2^ 0.723), ANFIS-GA (*RMSE* 1.878 mg/l, *MAE* 1.397 mg/l, *R*^2^ 0.696) ,and ANFIS-PSO (*RMSE* 1.909 mg/l, *MAE* 1.490 mg/l, *R*^2^ 0.673) in the test stage.

**Table 4:** Training and test statistics of the models for BOD prediction — ANFIS-PSO for Gongreung Station

Model inputs	Training period			Test period		
	*RMSE*	*MAE*	*R*^2^	*RMSE*	*MAE*	*R*^2^
pH, EC, DO, WT	2.519	1.778	0.494	2.901	2.410	0.268
pH, EC, DO, WT, COD	1.483	1.209	0.782	1.710	1.472	0.755
pH, EC, DO, WT, COD, SS,	1.404	1.126	0.802	1.663	1.250	0.766
pH, EC, DO, WT, COD, SS, T-N	1.302	1.025	0.817	1.628	1.148	0.799
pH, EC, DO, WT, COD, SS, T-N, T-P	1.334	1.041	0.806	1.642	1.170	0.785
*Mean*	1.608	1.236	0.740	1.909	1.490	0.673

**Table 5:** Training and test statistics of the models for BOD prediction — ANFIS-GA for Gongreung Station

Model inputs	Training period			Test period		
	*RMSE*	*MAE*	*R*^2^	*RMSE*	*MAE*	*R*^2^
pH, EC, DO, WT	2.503	1.737	0.501	2.835	2.266	0.333
pH, EC, DO, WT, COD	1.445	1.182	0.799	1.703	1.236	0.769
pH, EC, DO, WT, COD, SS,	1.392	1.024	0.813	1.642	1.215	0.785
pH, EC, DO, WT, COD, SS, T-N	1.221	0.939	0.823	1.596	1.113	0.803
pH, EC, DO, WT, COD, SS, T-N, T-P	1.261	0.970	0.817	1.614	1.155	0.792
*Mean*	1.564	1.170	0.751	1.878	1.397	0.696

**Table 6:** Training and test statistics of the models for BOD prediction — ANFIS-SCA for Gongreung Station

Model inputs	Training period			Test period		
	*RMSE*	*MAE*	*R*^2^	*RMSE*	*MAE*	*R*^2^
pH, EC, DO, WT	2.008	1.282	0.664	2.616	2.130	0.367
pH, EC, DO, WT, COD	1.332	0.989	0.808	1.587	1.146	0.799
pH, EC, DO, WT, COD, SS,	1.247	0.969	0.824	1.549	1.139	0.804
pH, EC, DO, WT, COD, SS, T-N	1.198	0.903	0.840	1.497	1.020	0.828
pH, EC, DO, WT, COD, SS, T-N, T-P	1.236	0.927	0.809	1.523	1.049	0.817
*Mean*	1.404	1.014	0.789	1.754	1.297	0.723

**Table 7:** Training and test statistics of the models for BOD prediction — ANFIS-MPA for Gongreung Station

Model inputs	Training period			Test period		
	*RMSE*	*MAE*	*R*^2^	*RMSE*	*MAE*	*R*^2^
pH, EC, DO, WT	1.994	1.245	0.683	2.457	2.064	0.411
pH, EC, DO, WT, COD	1.243	0.940	0.825	1.494	0.941	0.814
pH, EC, DO, WT, COD, SS,	1.054	0.918	0.835	1.453	0.894	0.826
pH, EC, DO, WT, COD, SS, T-N	1.028	0.881	0.848	1.403	0.844	0.843
pH, EC, DO, WT, COD, SS, T-N, T-P	1.037	0.896	0.839	1.437	0.872	0.834
*Mean*	1.271	0.976	0.806	1.649	1.123	0.746

Table 8 sums up the training and testing results of the MARS method for the Gyeongan Station. Like the Gongreung Sation, here, also the COD has the highest effect on BOD. Considering COD in the model inputs respectively improves the *RMSE*, *MAE*, and *R*^2^ by 7.3%, 9.9%, and 313% in the test stage, while the accuracy of MARS model slightly decreases in BOD prediction by adding T-P. The best accuracy was obtained from the model with pH, EC, DO, WT, COD, SS, and T-N inputs with the lowest *RMSE* (1.381 mg/l) and *MAE* (1.120 mg/l) and the highest *R*^2^ (0.786) in the test stage.

**Table 8:** Training and test statistics of the models for BOD prediction — MARS for Gyeongan Station

Model inputs	Training period			Test period		
	*RMSE*	*MAE*	*R*^2^	*RMSE*	*MAE*	*R*^2^
pH, EC, DO, WT	1.417	1.072	0.339	1.512	1.382	0.173
pH, EC, DO, WT, COD	0.846	0.633	0.756	1.402	1.245	0.714
pH, EC, DO, WT, COD, SS,	0.834	0.619	0.767	1.389	1.237	0.730
pH, EC, DO, WT, COD, SS, T-N	0.823	0.604	0.777	1.381	1.120	0.786
pH, EC, DO, WT, COD, SS, T-N, T-P	0.827	0.615	0.772	1.384	1.226	0.738
*Mean*	0.949	0.709	0.682	1.414	1.262	0.622

Tables 9, 10, 11 and 12 report the training and test results of the ANFIS-PSO, ANFIS-GA, ANFIS-SCA, and ANFIS-MPA in predicting BOD of Gyeongan Station, respectively. Similar to the Gongreung Station, the 4th input scenario (pH, EC, DO, WT, COD, SS, T-N) has the best accuracy in training and test stages; the lowest *RMSE* and *MAE* values are 0.730 mg/l and 0.464 mg/l for the ANFIS-PSO, 0.657 mg/l and 0.466 mg/l for the ANFIS-GA, 0.523 mg/l and 0.402 mg/l for the ANFIS-SCA, and 0.490 mg/l and 0.374 mg/l for the ANFIS-MPA in the test stage. Considering COD in the model inputs considerably improves the accuracy of hybrid ANFIS methods in BOD prediction, for example, *RMSE* decreases from 1.449 to 0.775 mg/l for the ANFIS-PSO, from 1.352 to 0.657 mg/l for the ANFIS-GA, from 1.119 to 0.523 mg/l for the ANFIS-SCA, and from 1.094 to 0.490 mg/l for the ANFIS-MPA in the test stage. Considering all input scenarios, the ranges of the *RMSE*, *MAE*, and *R*^2^ are from 1.449 mg/l, 1.184 mg/l, and 0.205 to 0.730 mg/l, 0.464 mg/l, and 0.809 for the ANFIS-PSO; from 1.352 mg/l, 1.090 mg/l, and 0.243 to 0.657 mg/l, 0.466 mg/l, and 0.834 for the ANFIS-GA; from 1.119 mg/l, 1.033 mg/l, and 0.387 to 0.523 mg/l, 0.402 mg/l, and 0.858 for the ANFIS-SCA; and from 1.094 mg/l, 0.945 mg/l, and 0.434 to 0.490 mg/l, 0.374 mg/l, and 0.874 for the ANFIS-MPA, respectively. It is clear from Tables 7, 8, 9, 10 and 11 that the best ANFIS-MPA model comprising 4th input scenario (pH, EC, DO, WT, COD, SS, T-N) outperforms the other hybrid models in predicting BOD of Gyeongan Station in the test stage; improvement in *RMSE* accuracy is by 33%, 25%, and 6.3% compared to the ANFIS-PSO, ANFIS-GA, and ANFIS-SCA models, respectively. Furthermore, according to the average statistics, the ANFIS-MPA has the lowest *RMSE* (0.620 mg/l) and *MAE* (0.497 mg/l) and the highest *R*^2^ (0.780) and its accuracy is followed by the ANFIS-SCA (*RMSE* 0.649 mg/l, *MAE* 0.533 mg/l, *R*^2^ 0.757), ANFIS-GA (*RMSE* 0.812 mg/l, *MAE* 0.611 mg/l, *R*^2^ 0.705), and ANFIS-PSO (*RMSE* 0.900 mg/l, *MAE* 0.624 mg/l, *R*^2^ 0.664) in the test stage.

**Table 9:** Training and test statistics of the models for BOD prediction — ANFIS-PSO for Gyeongan Station

Model inputs	Training period			Test period		
	*RMSE*	*MAE*	*R*^2^	*RMSE*	*MAE*	*R*^2^
pH, EC, DO, WT	1.295	0.972	0.447	1.449	1.184	0.205
pH, EC, DO, WT, COD	0.725	0.535	0.827	0.775	0.487	0.767
pH, EC, DO, WT, COD, SS,	0.704	0.513	0.838	0.779	0.496	0.766
pH, EC, DO, WT, COD, SS, T-N	0.687	0.509	0.844	0.730	0.464	0.809
pH, EC, DO, WT, COD, SS, T-N, T-P	0.700	0.509	0.838	0.765	0.487	0.783
*Mean*	0.822	0.608	0.759	0.900	0.624	0.664

**Table 10:** Training and test statistics of the models for BOD prediction — ANFIS-GA for Gyeongan Station

Model inputs	Training period			Test period		
	*RMSE*	*MAE*	*R*^2^	*RMSE*	*MAE*	*R*^2^
pH, EC, DO, WT	1.274	0.945	0.465	1.352	1.090	0.243
pH, EC, DO, WT, COD	0.627	0.506	0.840	0.696	0.492	0.804
pH, EC, DO, WT, COD, SS,	0.599	0.501	0.846	0.685	0.516	0.825
pH, EC, DO, WT, COD, SS, T-N	0.570	0.445	0.855	0.657	0.466	0.834
pH, EC, DO, WT, COD, SS, T-N, T-P	0.582	0.497	0.847	0.672	0.493	0.817
*Mean*	0.730	0.579	0.771	0.812	0.611	0.705

**Table 11:** Training and test statistics of the models for BOD prediction — ANFIS-SCA for Gyeongan Station

Model inputs	Training period			Test period		
	*RMSE*	*MAE*	*R*^2^	*RMSE*	*MAE*	*R*^2^
pH, EC, DO, WT	0.995	0.715	0.691	1.119	1.033	0.387
pH, EC, DO, WT, COD	0.588	0.402	0.888	0.540	0.414	0.838
pH, EC, DO, WT, COD, SS,	0.566	0.369	0.897	0.530	0.407	0.849
pH, EC, DO, WT, COD, SS, T-N	0.509	0.338	0.917	0.523	0.402	0.858
pH, EC, DO, WT, COD, SS, T-N, T-P	0.529	0.347	0.910	0.533	0.410	0.852
*Mean*	0.637	0.434	0.861	0.649	0.533	0.757

**Table 12:** Training and test statistics of the models for BOD prediction — ANFIS-MPA for Gyeongan Station

Model inputs	Training period			Test period		
	*RMSE*	*MAE*	R^2^	*RMSE*	*MAE*	R^2^
pH, EC, DO, WT	0.963	0.634	0.694	1.094	0.945	0.434
pH, EC, DO, WT, COD	0.499	0.324	0.918	0.516	0.397	0.859
pH, EC, DO, WT, COD, SS,	0.464	0.298	0.927	0.505	0.389	0.865
pH, EC, DO, WT, COD, SS, T-N	0.435	0.279	0.935	0.490	0.374	0.874
pH, EC, DO, WT, COD, SS, T-N, T-P	0.449	0.282	0.933	0.494	0.381	0.869
*Mean*	0.562	0.363	0.881	0.620	0.497	0.780

Table 13 gives the *t*-test outcomes of the best hybrid ANFIS models in BOD prediction for both stations. The statistics were computed considering significance level of 5% (two-tailed test). Higher absolute *t*-statistics (*t*-stat) than the critical one means that there is no significant difference between the means of computed and observed data. The model with the highest *t-*stat has the best robustness. It is apparent from Table 12 that the ANFIS-MPA has higher statistics (10.716 and 8.114) compared to the other models in Gongreung and Gyeongan stations, while the ANFIS-PSO has the lowest statistics among the hybrid ANFIS models.

**Table 13:** *t*-test of the models applied for BOD prediction

	MARS	ANFIS-PSO	ANFIS-GA	ANFIS-SCA	ANFIS-MPA
Gongreung Station					
*t*-stat	−6.277	−7.565	−8.101	−9.422	−10.716
*p*-value	3.067E−09	2.800E−12	1.279E−13	4.598E−17	1.446E−20
*t*-critical	1.975	1.975	1.975	1.975	1.975
Gyeongan Station					
*t*-stat	−2.095	−4.079	−4.244	−4.237	−8.114
*p*-value	3.749 E−03	3.155E−05	3.432E−05	3.548E−05	6.216E−14
*t*-critical	1.973	1.973	1.973	1.973	1.973

**Fig. 9:**
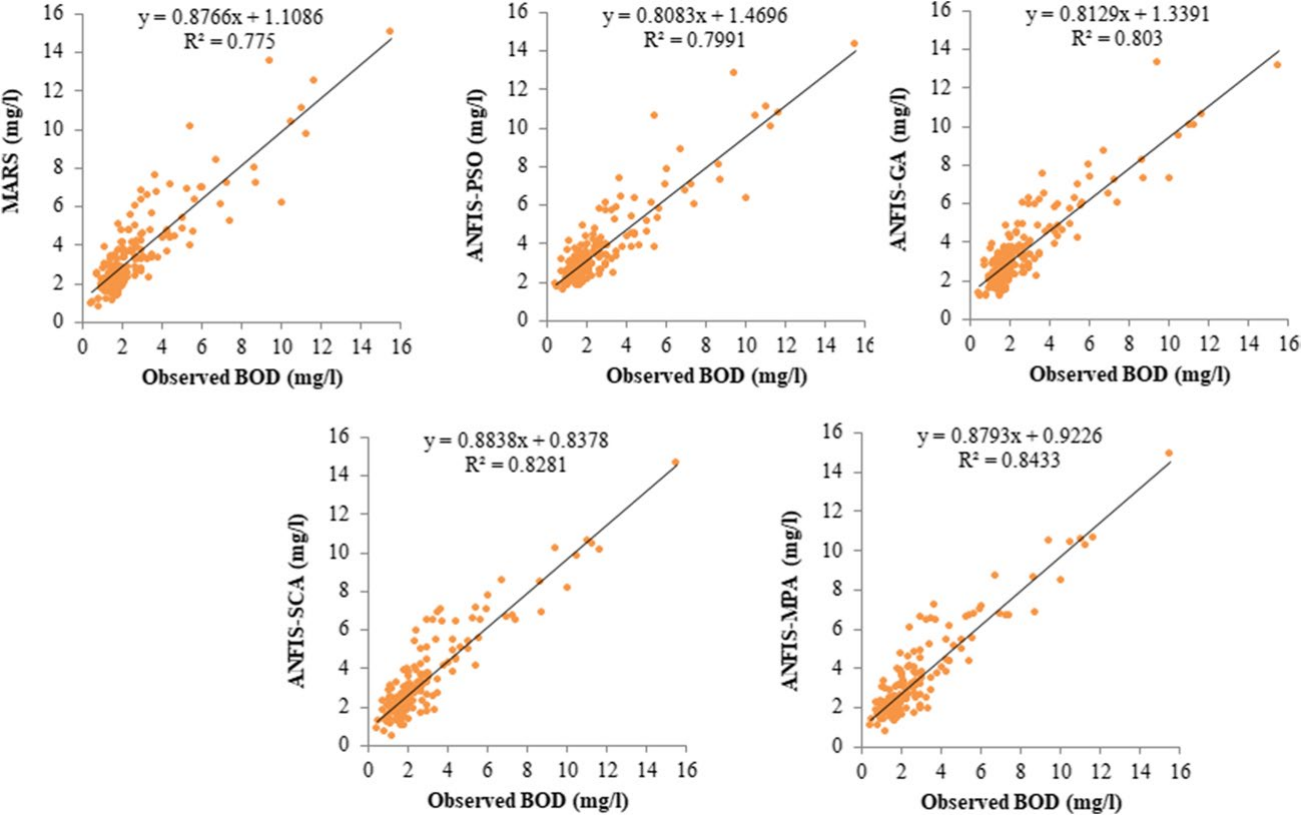
Scatterplots of the observed and predicted BOD by different models in the test period using the best input combination — Gongreung Station

**Fig. 10:**
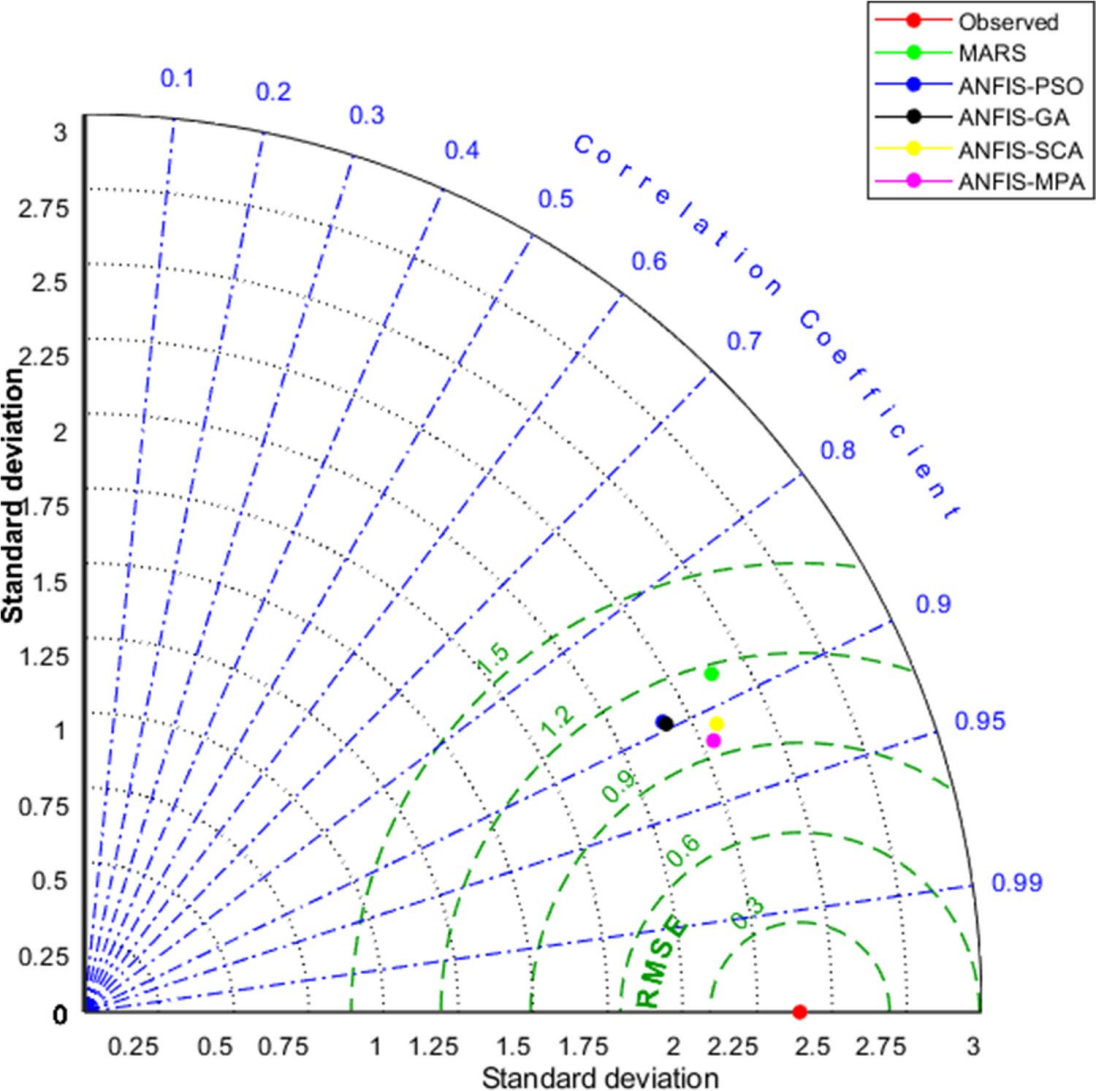
Taylor diagrams of the predicted BOD by different models in the test period using the best input combination — Gongreung Station

**Fig. 11:**
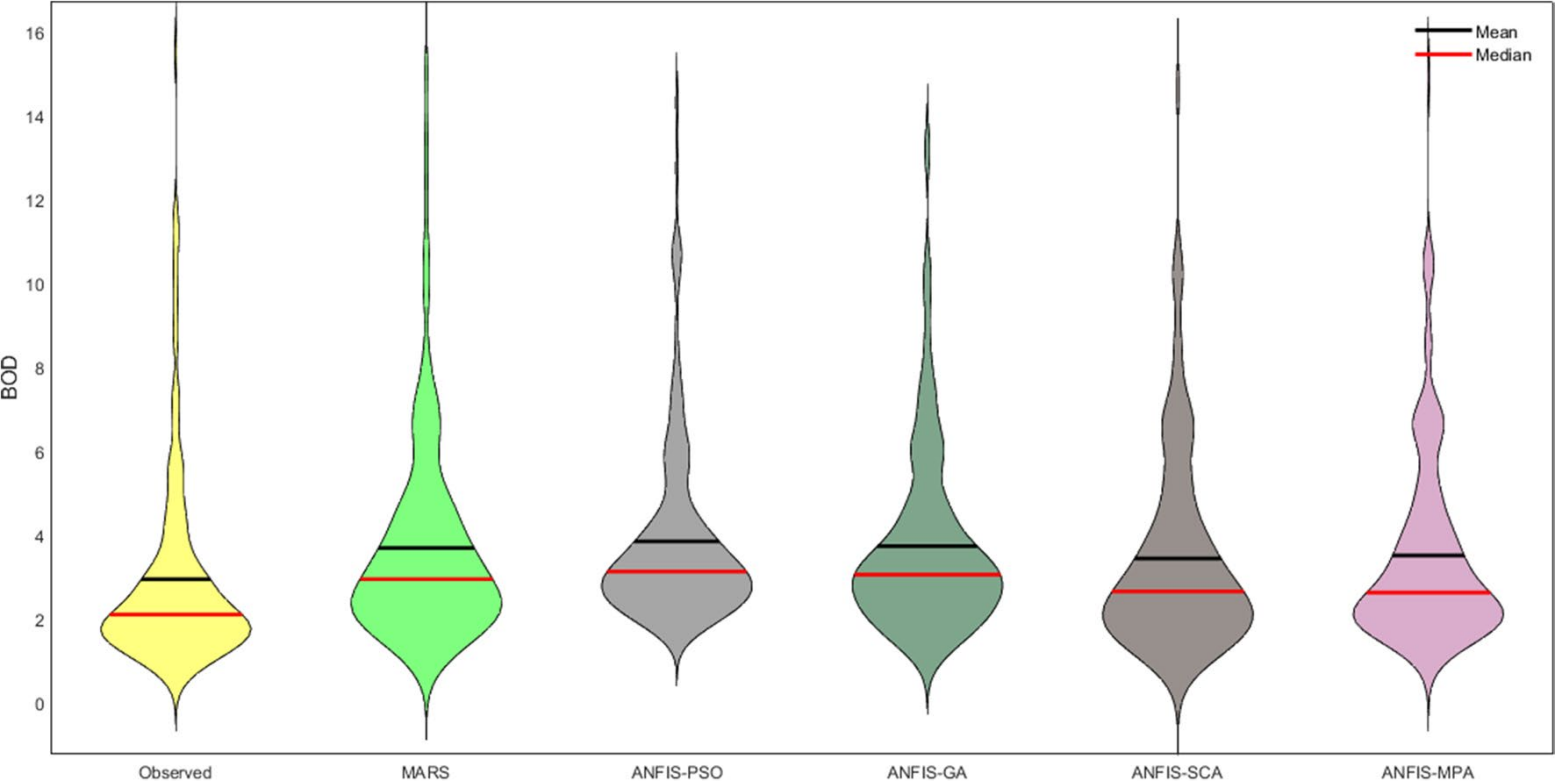
Violin charts of the predicted BOD by different models in the test period using the best input combination — Gongreung Station

The models were further compared with respect to their computational speed in training, and times in minutes were provided in Table 14. The models’ simulations were performed in the MATLAB environment (MATLAB R2017b) using a computer having Windows 10 (64 bit) with an Intel(R) Core(TM) i5-10500 CPU @ 3.10 GHz processor with 16 GB RAM. All input combinations were considered in this comparison. Table 14 clearly reports that the ANFIS-MPA has the fast speed and followed by the ANFIS-SCA, ANFIS, GA, and ANFIS-PSO among the hybrid ANFIS models. As expected, MARS model is faster than the hybrid ANFIS models because of having less complex structure. Figures 9–11

**Table 14:** Computational time of the reported models (in minutes)

Models	pH, EC, DO, WT	pH, EC, DO, WT, COD	pH, EC, DO, WT, COD, SS,	pH, EC, DO, WT, COD, SS, T-N	pH, EC, DO, WT, COD, SS, T-N, T-P	Mean time
MARS	0.1038	0.1085	0.1118	0.1185	0.01227	0.0910
ANFIS-PSO	0.1786	0.1826	0.1935	0.1956	0.1984	0.1897
ANFIS-GA	0.1673	0.1721	0.1758	0.1825	0.1877	0.1771
ANFIS-SCA	0.1609	0.1647	0.1683	0.1728	0.1754	0.1684
ANFIS-MPA	0.1531	0.1576	0.1598	0.1635	0.1676	0.1603

**Fig. 12:**
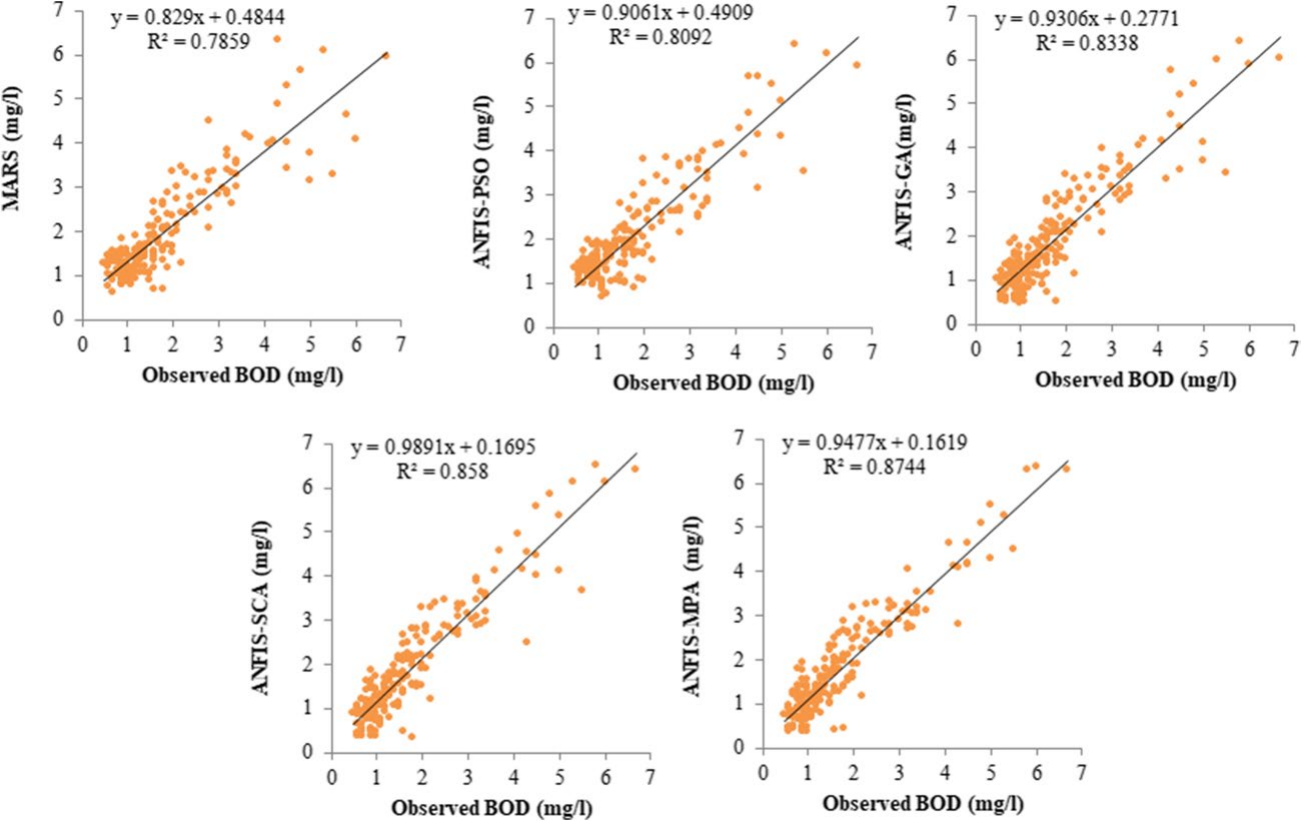
Scatterplots of the observed and predicted BOD by different models in the test period using the best input combination — Gyeongan Station

**Fig. 13:**
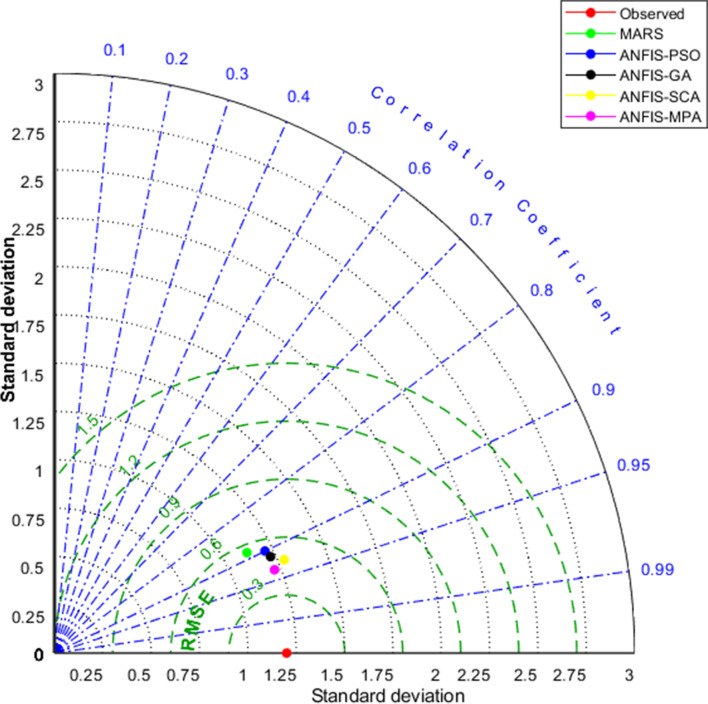
Taylor diagrams of the predicted BOD by different models in the test period using the best input combination — Gyeongan Station

**Fig. 14:**
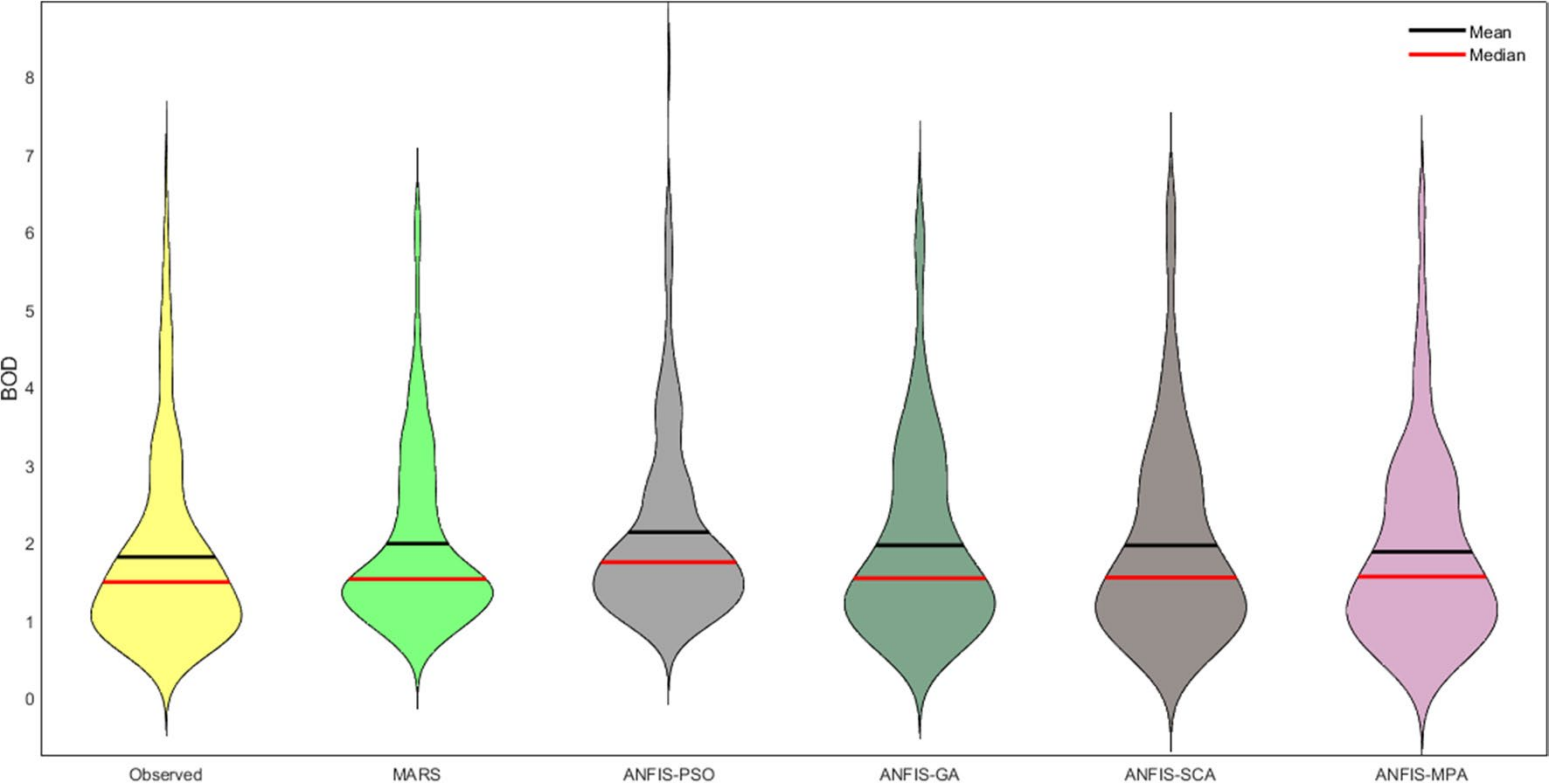
Violin charts of the predicted BOD by different models in the test period using the best input combination — Gyeongan Station

**Fig. 15:**
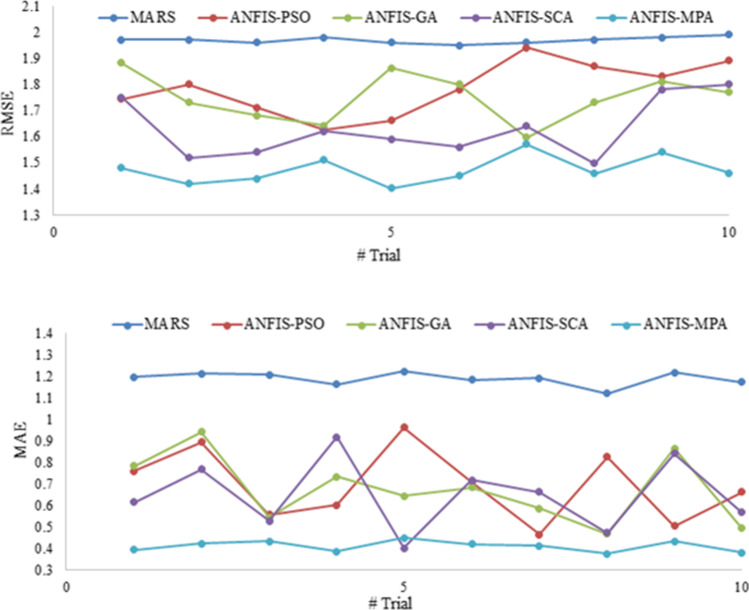
Stability of different models using 10 trials and *RMSE* and *MAE* metrics for Gongreung Station

**Fig. 16:**
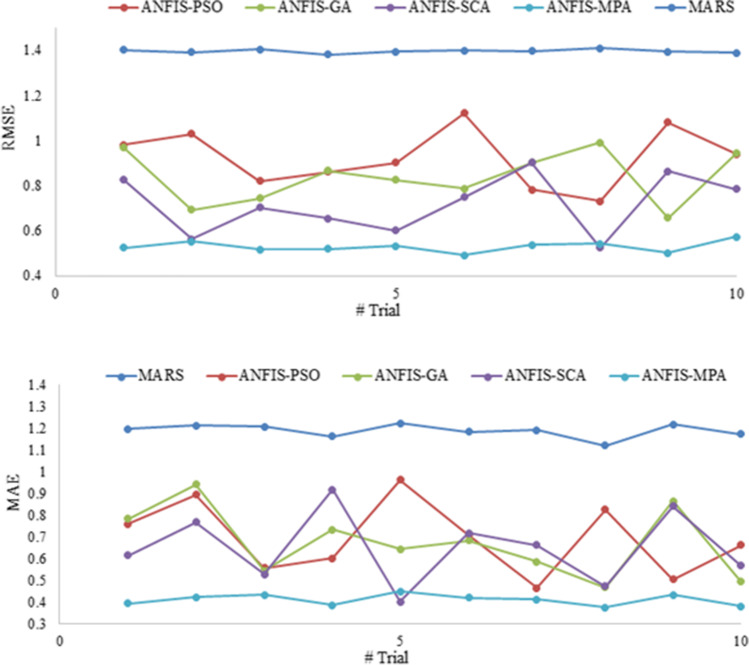
Stability of different models using 10 trials and *RMSE* and *MAE* metrics for Gyeongan Station

Figures 9 and 12 illustrate the scatterplots of observed and predicted BOD by the best MARS and hybrid ANFIS models in the test stage of Gongreung and Gyeongan stations. It is clear that the ANFIS-MPA has the least scattered predictions with the highest *R*^2^ followed by the ANFIS-SCA model in both stations. The best models are visually compared via Taylor diagrams in Figs. 10 and 13 based on *RMSE*, standard deviation, and correlation criteria. It is apparent from the diagrams that the ANFIS-MPA has the highest correlation and lowest square error in predicting the BOD of both stations. Figures 11 and 14 compare the distributions of the BOD predictions by the implemented models using violin charts. It is clearly observed from the charts that the mean and median and distribution shape of the ANFIS-MPA are more resembling those of the observed one (Figs. 11 and 14). The stability of the models was investigated by considering variation of *RMSE* and *MAE* statistics vs. different trials. Figures 15 and 16 respectively illustrate the variation of *RMSE* and *MAE* statistics for the Gongreung and Gyeongan stations in the test stage. It is clear from the both figures that the ANFIS-MPA has more stability compared to other hybrid models. For example, the *RMSE* of ANFIS-MPA ranges 1.4–1.6 while the ranges of the other models are about 1.5–2 for the Gongreung Station. MARS also has a high stability because of having less parameters, but it has the least accuracy.

## Discussion

By the presented study, the viabilities of four different hybrid ANFIS models were investigated to determine the best prediction model for BOD water quality parameter. First, MARS method was applied to investigate the best input scenario. Then, hybrid ANFIS methods were also applied to the same scenarios to see if MARS model is applicable for deciding the best scenario in predicting BOD. The outcomes of the hybrid ANFIS methods were found to be consistent with the trend of MARS results (e.g., the best accuracy was obtained from the 4th input scenario, while the 1st was the worst one). This implies that the MARS can be successfully applied in determination of the best input combination.

The outcomes of the MARS and hybrid ANFIS methods indicated that the COD input parameter has a considerable effect on BOD; improvement in *RMSE*, *MAE*, and *R*^2^ of ANFIS-MPA is by 39%, 54%, and 98% for Gongreung Station and by 52%, 58%, and 98% for Gyeongan Station, respectively. These results have direct dial with the study of Kim et al. (2020) in which same datasets were applied, and they found that the considering COD as input improves the accuracy of Deep Echo State Network (Deep ESN) by 38% and 80% for Gongreung and by 45% and 49% with respect to *RMSE* and *R*^2^ in BOD prediction in the test stage, respectively. Han and Qiao (2012) also previously reported the considerable influence of COD on BOD parameter. Comparison of the hybrid ANFIS methods indicated that the ANFIS-MPA offered superior performance in BOD prediction in all input scenarios. It improved the *RMSE* accuracy of the ANFIS-PSO, ANFIS-GA, and ANFIS-SCA models in BOD prediction by 13.8%, 12.1%, and 6.3% for the Gongreung Station and by 33%, 25%, and 6.3% for Gyeongan Station in the test stage, respectively. The hybrid ANFIS methods seem to be more successful in mapping BOD in Gongreung compared to Gyeongan (e.g., the *R*^2^ of the best ANFIS-MPA models respectively are 0.843 and 0.874). One reason for this can be higher skewness of the EC, SS, and BOD in both training and test datasets of Gongreung Station compared to those of the Gyeongan. SS as an important water quality parameter has a very high skewed distribution implying the chaotic structure of this data which was also previously reported by Adnan et al. (2021, 2022).

The outcomes were compared with the existing literature for the validation of the presented study. Khatri et al. (2019) applied ANN for predicting the effluent parameters of Jamnagar treatment plant in India, and they obtained correlation coefficient of 0.74 for BOD parameter. Sharafati et al. (2020) used AdaBoost regression, gradient boost regression, and random forest regression to predict the effluent quality parameters including BOD_5_, and they obtained correlation coefficient of 0.9 for BOD parameter. Kim et al. (2020) used deep ESN, gradient boosting regression tree (GBRT), extreme learning machine (ELM), and random forest (RF) for BOD prediction, and they obtained correlation coefficient of 0.892–0.924, 0.854–0.911, 0.890–0.915, and 0.868–0.918 for the best deep ESN, ELM, GBRT, and RF in the test stage, respectively. In the presented study, the correlation coefficients of 0.918 and 0.935 were obtained from the best ANFIS-MPA in BOD prediction for Gongreung and Gyeongan stations, respectively.

## Conclusions

In this study, the ability of four hybrid neuro-fuzzy models were investigated in predicting BOD using various input combinations composed of pH, EC, DO, WT, COD, SS, T-N, and T-P obtained from two stations, South Korea. MARS method was implemented in order to determine the optimal input combination and observed that this method can be successfully used for this purpose in predicting BOD as an important water quality parameter. The outcomes of the MARS and hybrid ANFIS methods indicated that the models with pH, EC, DO, WT, COD, SS, and T-N inputs offer the best accuracy, while the pH, EC, DO, and WT inputs provide the least performance in both stations. Comparison of the hybrid methods revealed that the ANFIS-MPA model performs superior to the other hybrid models in predicting BOD in both stations. The accuracy ranks of the compared methods were found as ANFIS-MPA > ANFIS-SCA > ANFIS-GA > ANFIS-PSO. The ANFIS-MPA improved the *RMSE* accuracy of ANFIS-PSO, ANFIS-GA, and ANFIS-SCA models by 13.8%, 12.1%, and 6.3% for Gongreung Station and by 33%, 25%, and 6.3% for Gyeongan Station in the test stage, respectively. Comparison with the previous literature showed the applicability of ANFIS-MPA model in BOD prediction.

The input parameters used in this study can be directly measured in the field using small equipment. However, biochemical oxygen demand cannot be directly measured but it can be indirectly determined by incubating at 20 °C during 5 days. Therefore, the hybrid ANFIS-MPA model can be used as a useful tool in predicting BOD using easily measured parameters and this is more economical and time-saving procedure.

## Data Availability

Research data is freely available on the website provided in the case study section.
